# Migration analysis of a metaphyseal-anchored short femoral stem in cementless THA and factors affecting the stem subsidence

**DOI:** 10.1186/s12891-019-2980-7

**Published:** 2019-12-12

**Authors:** Michael O. Schaer, Michael Finsterwald, Iris Holweg, Dimitris Dimitriou, Alexander Antoniadis, Naeder Helmy

**Affiliations:** 1 Department for Orthopedic Surgery and Traumatology, Buergerspital Solothurn, Schoengruenstrasse 42, 4500 Solothurn, Switzerland; 2 Department for Orthopedic Surgery and Traumatology, Inselspital, University of Bern, Bern, Switzerland

**Keywords:** Subsidence, Migration, Short-stem hip implant, EBRA, Cementless short-stem

## Abstract

**Background:**

Early femoral stem subsidence following a cementless THA is correlated with aseptic loosening of the femoral component. The short femoral stems allow bone sparing and implantation through a minimally invasive approach; however, due to their metaphyseal anchoring, they might demonstrate different subsidence pattern than the conventional stems.

**Methods:**

In this prospective single-center study, a total of 68 consecutive patients with an average age of 63 years, and a minimum follow-up of 5 years following a cementless THA with a metaphyseal-anchored short femoral stem were included. The femoral stem subsidence was evaluated using “Ein Bild Roentgen Analyse” (EBRA).

**Results:**

Average stem migration was 0.96 +/− 0.76 mm at 3 months, 1.71 +/− 1.26 mm at 24 months, and 2.04+/− 1.42 mm at last follow-up 60 months postoperative. The only factor that affected migration was a stem size of 6 or more (r^2^ = 5.74; *p* = 0.039). Subdivision analysis revealed, that only in females migration appeared to be affected by stem size irrespective of weight but not in men (female stem size of 6 or more vs. less (Difference = − 1.48 mm, R^2^ = 37.5; *p* = 0.001). Migration did not have an impact on clinical outcome measures.

**Conclusions:**

The examined metaphyseal-anchored short femoral stem showed the highest subsidence within the first 3 months postoperative, the implant began to stabilize at about 24 months but continued to slowly migrate with average total subsidence of 2.04 mm at 5 years following the THA. The amount of stem subsidence was not associated with worse clinical outcomes such as HHS, patient satisfaction, or pain.

## Background

Total hip arthroplasty (THA) is considered a highly successful procedure in providing pain relief, restoring hip function and improving the quality of life in patients suffering from end-stage hip osteoarthritis [1]. According to current estimations, 15% of THA are performed in active individuals, younger than 60 years [2] and this number is expected to increase in the near future [3].

Recent evidence suggests that young, active patients undergoing a THA might have a higher risk of implant failure and subsequent revision surgery [4]. Therefore, within this population, an effective THA should aim in preserving the metaphyseal bone, providing feasible femoral revision options and allowing easier implantation with less invasive procedures. Although conventional, uncemented stems have shown excellent implant survivorship and long-term outcomes [5], they might be associated with a reduction of trochanteric bone stock and thigh pain due to impingement with the diaphyseal femoral cortex [6]. Short cementless femoral components were developed to preserve metaphyseal bone through proximal load transfer and facilitate the femoral stem implantation through minimally invasive approaches. Despite the excellent short- to midterm outcomes of several short stem designs [7, 8], it remains unclear whether all cementless short femoral stems can achieve an adequate stem fixation.

Since early stem migration might be associated with aseptic loosening of the femoral component [9], the goal of the current study was to evaluate the 5-year subsidence of a metaphyseal-anchored short femoral stem in cementless THA, and to correlate the stem subsidence with patient demographics, implant characteristics such as stem and head size, as well as clinical outcomes.

## Material and methods

### Study design and participants

The current study was approved by the local ethical committee (EKNZ 2017–00435). Each patient provided written informed consent before participation. This study was conducted entirely at the authors’ institution. From 03/2011 until 08/2012, all the patients presented in the clinic with symptomatic hip osteoarthritis waiting for a hip arthroplasty were considered as potential candidates for this study. The follow-up period ended at 09/2017, to achieve a minimum 5-year follow-up. Data was prospectively collected and retrospectively analyzed.

### Inclusion criteria

**Fig. 1:**
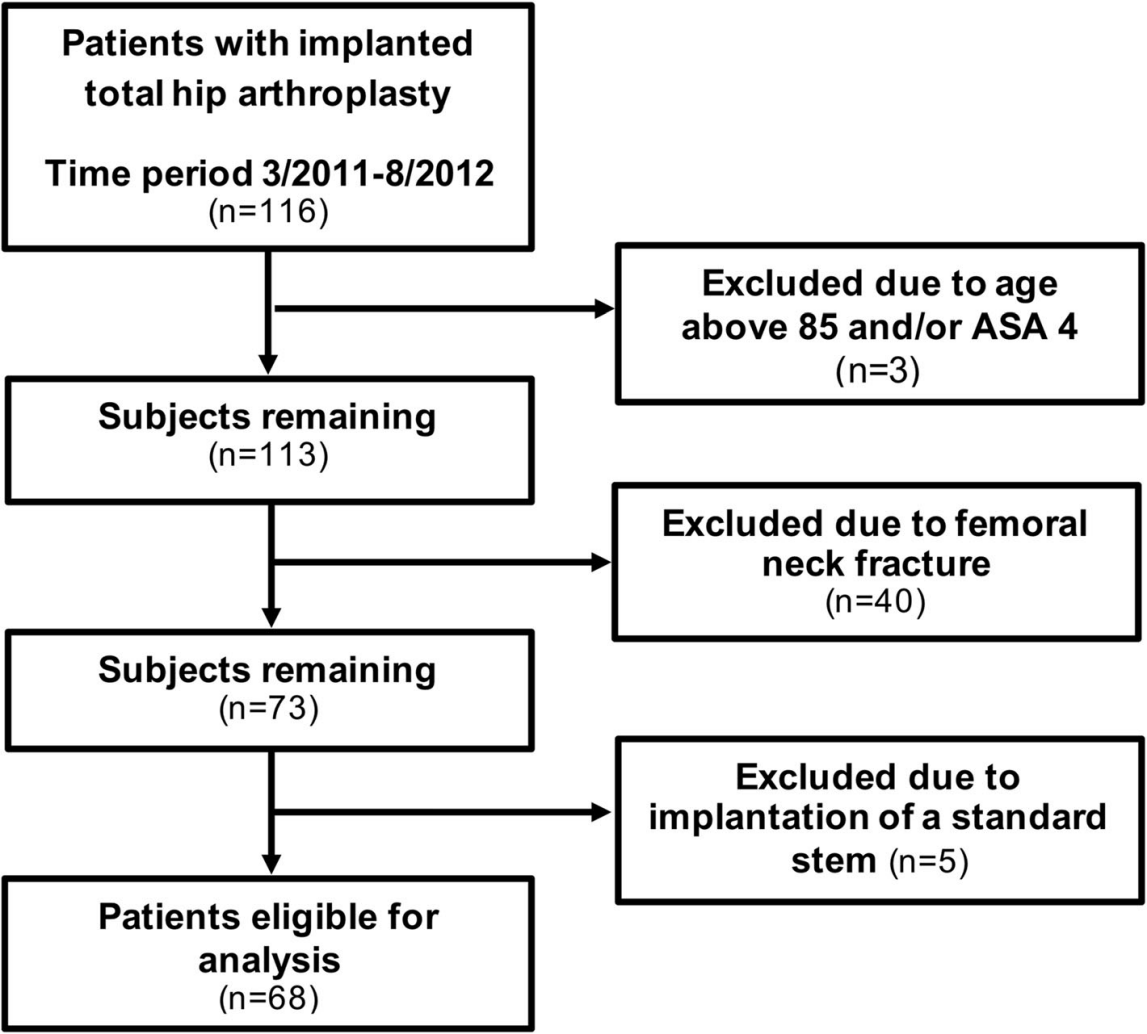
Study inclusion flow chart. ASA: American Society of Anesthesiologists score

The inclusion criteria were adult patients, between 18 and 85 years who received a primary THA and gave their informed consent. Exclusion criteria were: patients older than 85 years, patients undergoing revision surgery, American Society of Anesthesiologists score (ASA) higher than 3, and patient conditions that did not allow for implantation of a cementless short-stem femoral component, such as severe osteoporosis (Fig. 1). Furthermore, patients who suffered an intraoperative periprosthetic fracture were excluded from the study.

### Preoperative planning, surgery and implants

Preoperative templating was performed using the measurement templates provided by the company (Mathys, Bettlach, Switzerland) on calibrated standard x-rays in order to determine stem size and offset. All cases were performed via a standardized minimally invasive direct anterior approach in a supine position using the AMIS® Mobile Leg Positioner (Medacta International SA, Castel San Pietro, Switzerland) under spinal or general anesthesia. The procedure was performed by two experienced arthroplasty surgeons of our institution (> 100 THA/year). The implants used in the current study included a cementless acetabular component (RM Pressfit vitamys, Mathys, Switzerland) and a cementless femoral stem (optimys™, Mathys, Bettlach, Switzerland) with standard (24%) or lateral offset (77%) according to the preoperative planning (Table 1). The implant size ranged from 2 to 9. During surgery, the stem was aligned with respect to the femoral shaft according to the patients anatomy. In the implanted calcar guided stem, the entry point was determined in relation to the calcar. Version was determined by placing the stem parallel to the posterior wall which is a clear anatomic landmark in the direct anterior approach. If there was any doubt of complication (e.g intraoperative femur fracture), intraoperative fluoroscopy was performed.

**Table 1:** Patient demographics and stem characteristics

**Patient demographics**	**Value**
Age (Years)	63.3 (40, 83)
BMI (kg/m^2^)	29 (16, 43)
Gender	
• Male (n)	40 (59%)
• Female (n)	28 (41%)
Follow up (months)	65 (60, 83)
Side	
• Left (n)	40 (59%)
• Right (n)	28 (41%)
Dorr Classification (n)	
• Type A	46 (68%)
• Type B	22 (32%)
• Type C	0 (0%)
**Implant Characteristics**	**Value**
Head Size (mm)	
• 28	6 (8.8%)
• 32	31 (45.6%)
• 36	31 (45.6%)
Stem Offset	
• Standard	16 (24%)
• Lateral	52 (76%)
Stem size	
• 2	3 (4.4%)
• 3	7 (10.3%)
• 4	8 (11.8%)
• 5	16 (23.5%)
• 6	10 (14.7%)
• 7	15 (22.1%)
• 8	6 (8.8%)

Depicted are all the important patient demographics as well as characteristics of the hip stem. The values were given in average and range. n=68. BMI: Body mass index.

### Postoperative care

Beginning on the first postoperative day, all the patients followed a standardized physical therapy protocol with partial weight-bearing (half the body weight) for the first 2 weeks, followed by progression to full weight-bearing. Patients were discharged when able to mobilize for daily activities safely, pain controlled with oral medications, and were medically stable.

### Clinical and Radiographical evaluation

Preoperative X-ray assessments included Dorr’s classification [10] graded by two evaluators (MS and MF) from preoperative anteroposterior and lateral radiographs.

The patients were followed-up clinically and radiographically at 3, 6, 12, 24 and 60 months postoperatively. The clinical examination and HHS was performed by an independent to the study orthopedic surgeon of our clinic in a standardized matter. A standard AP pelvis and lateral radiograph of the hip were obtained during the first week following THA and at all follow-ups. The first postoperative radiograph was used as a baseline measurement.

### EBRA-FCA migration analysis

EBRA-FCA (Ein-Bild-Röntgen-Analyse-Femurkomponenten-Analyse) analysis is an established and accurate method in the measurement of the femoral component subsidence with a specificity of 100% and a sensitivity of 78% compared with roentgen stereophotogrammetric analysis (RSA) for the detection of migration of over 1 mm, and with a Cronbach’s coefficient alpha for interobserver reliability of 0.84 [11]. All 68 hips underwent analysis for axial stem subsidence using the EBRA-FCA software (Institute for Basic Engineering Sciences, University of Innsbruck, Innsbruck, Austria).

### Statistical analysis

A power analysis was performed to determine the appropriate sample size required to provide the statistical power to enable detection of a small difference between the migration rate of our cohort and the one of other uncemented short stems presented in the literature. A difference of 0.5 mm, i.e. half of the precision margin of the EBRA method, was considered as comparable. The power to detect a subsidence larger than 0.5 mm with the present study based on 66 patients amounts to 89% (alpha = 0.05, 2-sided).

Descriptive statistics used average and range to present the data. All parameters were tested with Shapiro-Wilk test for normality. When the criteria for normality were met, a two-tailed t-test was used. Otherwise, the Wilcoxon test was applied. A Pearson correlation was used to find potential relationships between stem migration and clinical outcomes such as HHS, patient satisfaction and pain at rest and under load. A stepwise multivariable regression analysis was applied to identify potential correlations between stem subsidence, patient demographics, implant characteristics, and clinical outcomes. All statistical analyses were performed with SAS version 9.4 (SAS Institute Inc., Cary, NC, USA).

## Results

### Participants’ characteristics

A total of 68 consecutive patients (Male: 28, Female: 40) with an average age of 63 years (range: 38–81 years) met the inclusion criteria of the current study (Table 1). Indications for THA were primary osteoarthritis in 62 cases (91%), secondary osteoarthritis in 4 cases (6%), congenital dysplasia in 1 case (1.5%), and osteonecrosis in 1 case (1.5%). No patient was lost during the 5-years follow-up.

### Complications

**Fig. 2:**
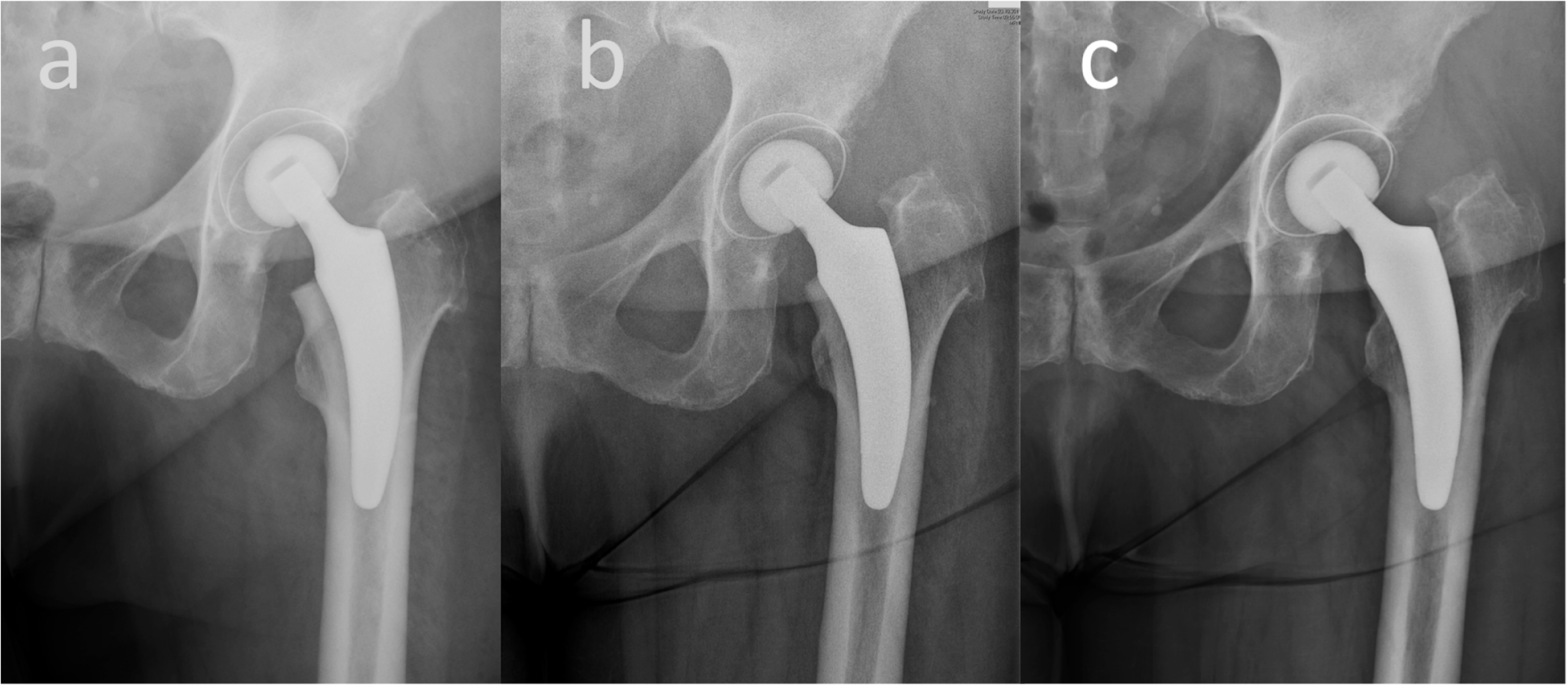
Aseptic loosening of the stem in one patient. The x-ray images of the case, in which an aseptic loosening was diagnosed is depicted. **a** Postoperative, **b** 3 months and **c** 6 months postoperative images are presented

At a minimum 5-year follow-up after THA, two stems (3%) had to be revised, one (1.5%) due to a periprosthetic fracture (Vancouver B2) following a fall, and one (1.5%) due to aseptic loosening of the stem (Fig. 2). No other complications were reported.

### EBRA-FCA migration analysis

**Fig. 3:**
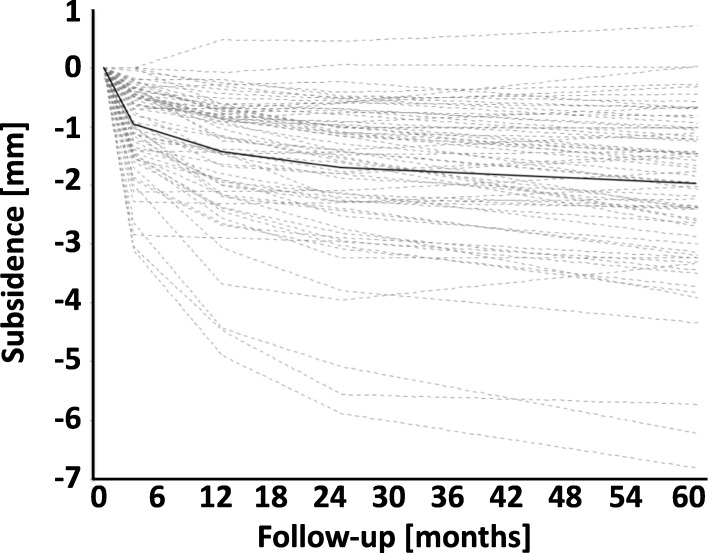
Subsidence up to 5 years postoperative. This graph showes the subsidence of all 68 hips up to 5 years postoperative. The average subsidence is shown with a continuous *black line*

**Fig. 4:**
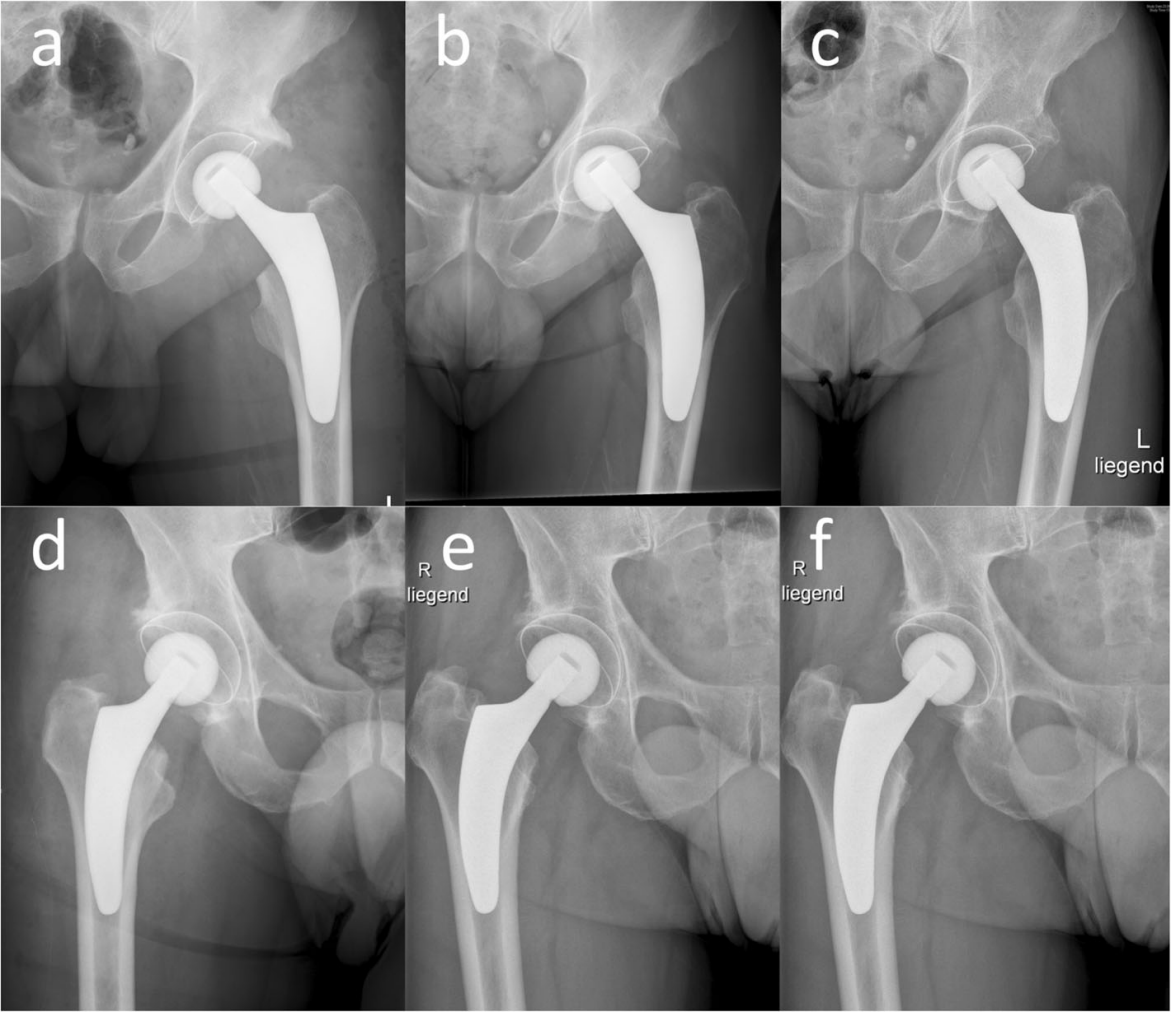
Representative cases without and with 3 mm of subsidence. In this figure, a case, in which no subsidence (**a**–**c**) and a case, in which 3 mm of subsidence (**d**–**f**) was seen up to 5 years postoperative are presented. Postoperative (**a** and **d**), 12 months postoperative (**b** and **e**) and 5 years postoperative (**c** and **f**) a-p x-rays are depicted

The average stem subsidence was 0.96 +/− 0.76 mm at 3 months, 1.43 +/− 1.07 mm at 12 months, 1.71 +/− 1.26 mm at 24 months, and 2.04 +/− 1.42 mm at the final follow-up, 60 months postoperative. The highest subsidence occurred during the first 3 months, the implant began to stabilize at about 24 months but continued to slowly subside until 5 years following the THA (Figs. 3 and 4).

### Patient factors affecting migration

The patient’s age did not have an impact on stem migration. Patients older than 65 years did not show a higher migration up to 60 months postoperative compared to patients younger than 65 years (Table 2). There was no difference with respect to migration at last follow up between male (2.22 +/− 1.02 mm) and female patients (1.80 +/− 1.63 mm)(*p* = 0.5). Patients weighing more than 75 kg did not show more subsidence compared to patients weighing less than 75 kg. The Dorr proximal femur morphology did not reveal an impact of the bone quality on subsidence at last follow-up (Table 2).

**Table 2:** Impact of different factors on amount of migration

**Patient demographics**	**Subsidence (mm)**						***p***-**Value**
*Age (years)*	*≤65*	2.04 (±1.4)	*> 65*	2.05 (±1.44)	–		0.76
*Gender*	*male*	2.22 (±1.02)	*female*	1.8 (±1.63)	–		0.53
*Weight (kg)*	*< 75 kg*	1.67 (±1.07)	*≥75*	2.2 (±1.52)	–		0.27
*BMI (kg/m*^*2*^*)*	*< 30*	2.16 (±1.6)	*≥30*	1.8 (±1.63)	–		0.6
*Dorr classification*	*Type A*	1.99 (±1.39)	*Type B*	2.16 (±1.49)	Type C	NA	0.62
**Implant Characteristics**	**Subsidence (mm)**						***p***-**Value**
*Offset*	*standard*	1.88 (±1.2)	*lateral*	1.48 (±1.73)	–		0.85
*Head Size (mm)*	*28*	1.44 (±0.76)	*32*	1.94 (±1.42)	*36*	2.26 (±1.5)	0.38
*Stem Size*							
*all patients*	*< 6*	1.53 (±0.9)	*≥6*	2.56 (±1.65)	–		< 0.05*
*females*	*< 6*	1.48 (±0.8)	*≥6*	2.97 (±0.8)	–		< 0.05*
*males*	*< 6*	1.61 (±1.0)	*≥6*	2.48 (±1.8)	–		0.221

BMI: (Body mass index).

NA: Not applicable.

*indicates statistically significant difference

This table compares the impact of different patient-specific factors and implant factors on the amount of migration. The values are given in average and range.

### Implant factors affecting migration

The two different stem offset options (standard and lateral) did not have an impact on stem subsidence (offset standard: 1.88 +/− 1.21 mm versus offset lateral: 1.48 +/− 1.73 mm; *p* = 0.845). Head size did not have an impact on stem migration (head size 28 (*n* = 6): 1.44 +/− 0.76 mm, head size 32 (*n* = 31): 1.94 +/− 1.42 mm, head size 36 (n = 31): 2.26 +/− 1.49 mm)(*p* = 0.382).

A stepwise multivariate analysis showed that the only factor that affected subsidence was the stem size (r^2^ = 5.74; *p* = 0.039). A stem size of ≥6 (the median implant size) showed a subsidence of 2.56 +/− 1.65 mm compared to stem sizes lower than 6 with 1.53 +/− 0.89 mm (*p* = 0.007) at the last follow-up. However, subdivision analysis revealed, that only in females migration appeared to be affected by stem size irrespective of weight but not in men (female stem size of ≥6 vs. lower than 6 (Difference = − 1.48 mm, R^2^ = 37.5; *p* = 0.001)).

### Clinical outcomes and stem migration

At the last follow-up, the amount of stem subsidence did not correlate with the HHS (r = − 0.04; *p* = 0.77), patient satisfaction (r = 0.01; *p* = 0.93) or pain under load (r = − 0.17; *p* = 0.16) (Table 3).

**Table 3:** Correlations

Clinical outcome	Subsidence (Correlation coefficient)	p-Value
Harris Hip Score	−0.04	0.77
Patient satisfaction	0.01	**0.93**
Pain at rest	0.10	0.42
Pain under load	−0.17	**0.16**

Minus indicates negative correlation.

*indicates statistically significant difference

Correlation between the amount of subsidence up to 5 years postoperative and clinical outcome measures such as Harris Hip Score (HHS), pain satisfaction score, pain under rest and pain under load.

## Discussion

Early stem subsidence following a cementless THA is correlated with aseptic loosening of the femoral component [9, 12–14]. Despite the excellent short- to midterm outcomes of several short stem designs [7, 8], it remains unclear whether all cementless short femoral stems could achieve an adequate stem fixation. The aim of the current study was to evaluate the 5-year subsidence of a metaphyseal-anchored short femoral stem in cementless THA and to correlate stem subsidence with patient demographics, implant characteristics, and clinical outcomes. The results of this study showed that the highest subsidence occurred during the first 3 months, the implant began to stabilize at about 24 months, but continued to slowly subside with an average total subsidence of 2.04 mm at 5 years following the THA. The stem subsidence was not significantly correlated with patient’s age, gender, weight or bone quality. The only implant factor that affected stem subsidence was stem size of 6 or more in females. The amount of stem subsidence was not associated with worse clinical outcomes.

The short femoral stems in cementless THA exhibit the highest subsidence during the first 3 months following THA, but subsidence might occur up to 5 years. Specifically, Freitag et al. [15] reported an average subsidence of 1.1 mm (range: -5 mm to 1.5 mm) in a different short stem design (Fitmore®, Zimmer Inc., Warsaw, Indiana, USA) up to the 5 year follow-up with the maximum subsidence occurring during the first 3 months following THA. However, Acklin et al. [16] observed a slightly lower average subsidence with the Fitmore short stem of 0.39 mm at 3 months which was stable until the 2-year follow-up. Brinkmann et al. [17] comparing the Metha stem (Aesculap AG, Tuttlingen, Germany) with the Nanos stem (Smith & Nephew plc, London, UK) reported subsidence of 1.96+/− 2.37 mm and 2.04 +/− 2.65 mm, at 1 year following THA, respectively, with most migration occurring also during the first 3 months. In accordance with the literature, we observed that the optimys metaphyseal-anchored stem subsidence was 0.96 +/− 0.76 mm at 3 months, 1.43 +/− 1.07 mm at 12 months, 1.71 +/− 1.26 mm at 24 months, and 2.04 +/− 1.42 mm at the final follow-up, 60 months postoperative. It showed the highest subsidence during the first 3 months with an average subsidence of 0.96 mm, began to stabilize at about 24 months but continued to slowly subside until 5 years following the THA. The data suggest that the optimys metaphyseal-anchored short stem in cementless THA exhibit similar subsidence pattern as other contemporary short stem designs.

Among all the different factors influencing subsidence, press-fit is believed to be one of the key factors [18, 19]. Even though the surgeon is guided by visual, sensory and auditory clues when inserting the femoral stem, finding a good balance between a perfect press-fit level and not fracturing the femur by the stem remains challenging. Even though, in our study femoral stems did not seem undersized when evaluating the postoperative x-rays, a discrete undersizing which may not be visible on x-rays could be responsible for the postoperative migration and settle-in of the femoral prosthesis. The stems migrate until they reach a firm press-fit level and therefore the rate of migration decreases over time. This assumption is supported by the fact, that in our study almost all stems showed some degree of initially pronounced migration but only one of 68 stems (1.5%) showed signs of aseptic loosening at the 5 year follow-up. Similar results were reported by Kutzner et al. [20], who showed initial migration but no aseptic loosening up to 2 years postoperative. Further studies are necessary to confirm that even though these uncemented short stems migrate postoperatively, they remain stable in the long term.

The uncemented short femoral stems might show similar subsidence with conventional straight femoral stems. Specifically, Ferguson et al. [21] in a randomized controlled trial comparing the subsidence of the Meta Fix conventional stem (Corin Group, Cirencester Gloucestershire, UK) with the short stem (MiniHip, Corin Group) reported an average subsidence of 0.62 +/− 0.56 mm and 0.26 +/− 0.38 mm, respectively, at the 2-year follow-up. McCalden et al. [22] reported a slightly higher, but statistically not significant subsidence in the SMF short stem (Smith & Nephew plc) compared to the Synergy conventional stem (Smith & Nephew plc) using RSA analysis (0.94 +/− 1.71 mm versus 0.32 +/− 0.45 mm, *p* = 0.66), 2 years following the THA. The result of the present study suggests that despite the metaphyseal-anchoring of the short femoral stem in cementless THA it might show a similar subsidence rate as the conventional straight femoral stem.

According to the literature, stem subsidence in several short stem design is affected by patient characteristics such as weight, BMI and age, whereas other stem designs stay unaffected from patient demographics. Kutzner et al. [20] investigating the same metaphyseal-anchoring stem as in our study (optimys, Mathys Ltd.) reported a significant influence of weight above 75 kg on mean axial migration at the 2-year follow-up. However, weight did not appear to influence stem subsidence when adjusted for age and gender. Stihlsen et al. [23] investigating the Vision-2000 stem (Depuy Orthopaedics Inc., Warsaw, Indiana, USA) found that body weight over 75 kg has a significant impact on stem subsidence at 2-year follow-up. In Fitmore (Zimmer Inc.) and Nanos (Smith & Nephew plc) short stem, BMI ≥30 kg/m^2^, age and weight did not influence the stem subsidence [15, 24]. Similarly, in our cohort body weight, BMI, age and gender did not have a statistically significant impact on the amount of stem subsidence.

The present study is the first to report a correlation between stem size and subsidence. Stem size ≥6 showed higher subsidence up to 5 years postoperative compared to stems < 6 in women only. One possible explanation might be that the surgeon did not chose a larger stem due to the fear of intraoperative periprosthetic fractures. Since women tend to need smaller implants than men, surgeons might be especially cautious when inserting bigger stem sizes in women and therefore do not achieve press-fit fixation in this cohort. In other implants such as the Nanos stem (Smith & Nephew plc), implant size did not influence the amount of migration [24].

In our cohort, postoperative stem migration did not have a negative impact on the clinical outcome or revision rate up to 5 years postoperative. This suggests that stem migration up to 2.04 +/− 1.42 mm at 5 years following THA is not clinically relevant in an uncemented metaphyseal-anchored short femoral stem.

The present study should be interpreted in light of its potential limitations. Although the RSA method is considered the gold standard for measuring the stem subsidence, it is nowadays rarely used due to the need for marker implantation and potential complications. The computer-assisted EBRA-FCA system is however an established and accurate method in measuring femoral component subsidence with a specificity of 100% and a sensitivity of 78% compared with the RSA for the detection of migration of over 1 mm [11]. Additionally, the current study investigated only the optimys short femoral stem. Although this design is similar to other short femoral implants available, our findings might not apply to other stem designs.

## Conclusion

The optimys short femoral stem in cementless THA hip implant showed the highest migration within the first 3 months postoperative, the implant began to stabilize at about 24 months, but continued to slowly migrate with an average total subsidence of 2.04 mm at 5 years following the THA. The stem subsidence was not significantly correlated with patient’s age, gender, weight or femoral bone quality assessed with the Dorr classification. The only implant factor that affected stem subsidence was stem size ≥6. However, this difference was only present in females. The amount of stem subsidence was not associated with worse clinical outcomes, in terms of HHS, patient satisfaction and pain.

## Data Availability

The datasets used and/or analyzed during the current study are available from the corresponding author on reasonable request.

## Abbreviations

ASA: American Society of Anesthesiologists score
BMI: Body Mass Index
EBRA-FCA: Ein-Bild-Röntgen-Analyse-Femurkomponenten-Analyse
HHS: Harris Hip Score RSA Roentgen Stereophotogrammetric Analysis
THA: Total Hip Arthroplasty
